# Disparities in health and health care in Myanmar

**DOI:** 10.1016/S0140-6736(15)00987-3

**Published:** 2015-11-21

**Authors:** Phyu Phyu Thin Zaw, Thant Sin Htoo, Ngoc Minh Pham, Karen Eggleston

**Affiliations:** a Department of Medical Research, Ministry of Health, Pyin Oo Lwin, Myanmar; b Myanmar Ministry of Health, Nay Pyi Taw, Myanmar; c Thai Nguyen University of Medicine and Pharmacy, Thai Nguyen City, Vietnam; d Stanford University Asia Health Policy Program, Stanford, CA 94305, USA

Myanmar (Burma) is undergoing a complex political and economic transformation, from a long civil war and military regime to a peace process and democratisation. Since 2011, the Myanmar Ministry of Health has started to rehabilitate the fragile health system, setting the goal of achieving universal health coverage by 2030.^1^ To achieve this target, Myanmar will have to face substantial challenges; arguably one of the most important difficulties is how to allocate limited health-care resources equitably and effectively.

Attention to the most vulnerable people would substantially improve national health outcomes. Myanmar's life expectancy at birth of 66·8 years and infant mortality of 62·0 years are the worst in southeast Asia. Persistent inequalities exist in health outcomes in Myanmar's seven states and seven regions.^2^ Residents of mountainous peripheral states suffer from remoteness, civil conflicts, and low socioeconomic development.^3^ For example, infant mortality is 94·2 per 1000 births and under-five mortality is 149·1 per 1000 births near the eastern border of Myanmar, where malaria and tuberculosis prevalence are also high.^4^ The gap in life expectancy at birth between the areas with the highest and lowest values within Myanmar is more than 11 years, almost as large as that between Myanmar and the USA.

Such disparities are not surprising when reliance on out-of-pocket financing is among the highest globally (81% of Myanmar's total health expenditures) because of no reliable health insurance system and the tight fiscal space for health. As an important step towards universal health coverage, the government increased health-care expenditures by 8·7 times from 2011 to 2015. However, resource allocation does not seem to be closely aligned with the goal of reducing health disparities.^5^ Conventional budget allocation, tied to population and infrastructure (figure^3^), gives disproportionately more resources to regions with better health, and fewer resources to several states with high health needs (as measured by infant mortality in the figure).

Crafting policies to mitigate rather than exacerbate health disparities needs professional and innovative leadership, which is one reason why Myanmar's physicians are protesting militarisation of the Ministry of Health through the Black Ribbon Movement. Development of a workable health policy to distribute resources equitably is not only crucial to improve the health status of the population—raising the average by prioritising the needs of the most vulnerable people—but also to build trust, to support peace and reconciliation, and to control the spread of drug-resistant strains of tuberculosis and malaria. These diseases fester in the border areas of Myanmar, and could develop into important regional public health concerns.

## Figures and Tables

**Figure:**
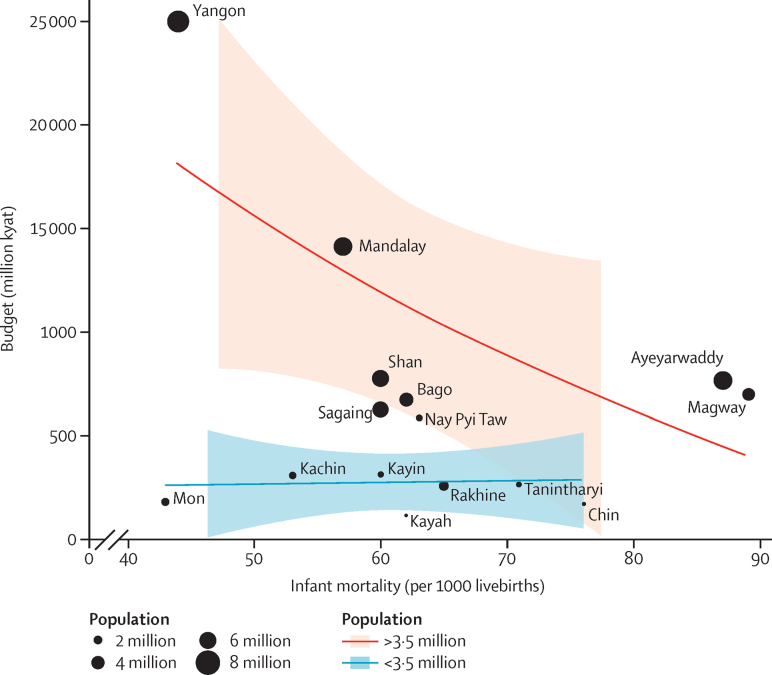
Health budgets for Myanmar's regions and states are not proportionate to health needs The lines are smooth curves fitted to the data by use of local regression and dots are roughly proportional to population size. Data taken from the Department of Population, Ministry of Immigration and Population.^3^
